# The Relationships Among Tinnitus Intensity, Complexity, Sound Intolerance, and Psychiatric Symptoms

**DOI:** 10.3390/jcm15166382

**Published:** 2026-08-18

**Authors:** Stefani Maihoub, Panayiota Mavrogeni, Aphrodite Mavrogenis, András Molnár

**Affiliations:** 1 Maihoub ENT Clinic, Aliakmona Street 16, 3117 Limassol, Cyprus; 2 Faculty of Health Sciences Doctoral School, University of Pécs, Vörösmarty Street 4, 7621 Pécs, Hungary; 3 Bajcsy–Zsilinszky Hospital, Maglódi út 89–91, 1106 Budapest, Hungary; 4 Opera Clinic, Protone Audio Kft., Lázár u. 4, 1065 Budapest, Hungary

**Keywords:** tinnitus intensity, pitch, tinnitus complexity, sound intolerance, depression, anxiety

## Abstract

**Objective**: This study investigated the associations between tinnitus-related features, psychological symptoms, and everyday functioning. **Material and Methods**: In this study, 293 patients with primary subjective tinnitus participated. Participants received otorhinolaryngological and audiological evaluations and completed the Tinnitus Handicap Inventory (THI), Beck Depression Inventory (BDI), and Generalised Anxiety Disorder-7 (GAD-7) questionnaires. **Results**: Significant positive correlations were observed between total THI scores and several tinnitus characteristics, including subjective loudness, pitch, complexity, and sound intolerance. Higher THI scores were also associated with higher depression and anxiety scores. Psychoacoustically matched tinnitus intensity was positively correlated with perceived tinnitus loudness; however, no significant association was found between overall THI scores and psychoacoustic tinnitus intensity. Longer tinnitus duration was positively correlated with both tinnitus loudness and participant age, while age was also positively associated with tinnitus loudness. Subjective tinnitus loudness, pitch, complexity, and sound intolerance were positively correlated with both BDI and GAD-7 scores. After applying the Bonferroni correction for multiple comparisons, the following correlations were no longer statistically significant: psychoacoustic tinnitus intensity with subjective tinnitus loudness and the THI Functional (*F*) subscale; tinnitus duration with subjective tinnitus loudness; subjective tinnitus loudness with BDI and GAD-7 scores; and age with subjective tinnitus loudness and tinnitus pitch. However, all remaining significant correlations were retained. In linear regression analyses, THI scores were independently associated with sound intolerance, tinnitus pitch, and BDI scores, while THI scores were also independently associated with BDI scores and sound intolerance in reciprocal models. **Conclusions**: In this single-centre, cross-sectional study of patients with tinnitus and without a control group, greater tinnitus handicap was associated with subjective tinnitus pitch, sound intolerance, and depressive symptoms. Subjective tinnitus loudness was positively associated with psychoacoustically matched tinnitus intensity before correction for multiple testing but was not retained after Bonferroni adjustment. Several subjective tinnitus characteristics remained associated with symptoms of depression and anxiety after adjustment for multiple comparisons. These findings describe associations within the studied population and should not be interpreted as evidence of causal relationships or generalised beyond similar clinical populations without further multicentre, longitudinal, controlled studies.

## 1. Introduction

Tinnitus refers to the perception of sound in the absence of an external acoustic stimulus [1]. The global prevalence of tinnitus is rising, with an annual incidence of about 1% and 14% of adults experiencing the condition [2]. Tinnitus can be classified based on potential underlying causes into primary and secondary cases. Its main types are subjective and objective tinnitus, with subjective primary tinnitus being the most common form [3]. Tinnitus can significantly diminish quality of life and is often linked to psychological symptoms like anxiety and depression [4]. The loudness of tinnitus appears to be significant, as suggested by both subjective reports and audiological assessments, such as tinnitus matching. Previous studies have shown weak correlations between tinnitus loudness and its impact on daily functioning, as measured by the Tinnitus Handicap Inventory (THI) [5]. Furthermore, previous findings have suggested no association between psychoacoustic tinnitus intensity measurements and the subjective perception of tinnitus loudness [6]. Another notable issue associated with tinnitus is the sound intolerance that many sufferers often report [7].

Previous investigations have shown conflicting results regarding subjectively reported tinnitus severities and psychoacoustically matched tinnitus intensities. For example, one study reported a significant correlation between the THI, visual analogue scale of annoyance, and tinnitus loudness matching [8]. However, another study found no correlation between THI scores and tinnitus loudness [5]. A different investigation found no connection between the loudness of sensation levels and the tinnitus severity reported by individuals [9]. Other findings indicated that the visual analogue scale for tinnitus loudness does not typically align with psychoacoustic measures of tinnitus loudness. This correlation was observed only in tinnitus patients with normal hearing [10]. Other results indicated weak to moderate correlations between tinnitus loudness matching and constrained loudness scaling, as well as a numeric rating scale [11].

Given the substantial effect of tinnitus on well-being and mental health, this study explored how different tinnitus features, including loudness, relate to quality of life and psychiatric symptoms. Despite its importance, there is still limited understanding of how these factors influence individual experiences with tinnitus.

Given previous evidence concerning tinnitus-related distress and psychological symptoms, the following hypotheses have been formulated:

**H1.**  *Increased subjective tinnitus characteristics, such as loudness, pitch, complexity, and sound intolerance, are positively correlated with tinnitus-related handicap (measured by the THI).*

**H2.**  *There is a positive association between greater tinnitus-related handicap and symptoms of depression (measured by the Beck Depression Inventory), as well as anxiety (measured by the Generalised Anxiety Disorder-7).*

**H3.**  *Psychoacoustically matched tinnitus intensity is positively correlated with subjective tinnitus loudness, but it does not necessarily correlate with the overall tinnitus-related handicap (THI).*

**H4.**  *Longer durations of tinnitus and older age correlate with higher tinnitus loudness and intensity, while increased age is linked to lower tinnitus frequency.*

**H5.**  *Characteristics of subjective tinnitus, such as loudness, pitch, complexity, and sound intolerance, are positively associated with symptoms of depression and anxiety.*

**H6.**  *Tinnitus characteristics and psychological symptoms independently predict the handicap associated with tinnitus in multivariable regression models.*

### Null Hypotheses

For each hypothesis, the null hypothesis asserts that no statistically significant association or predictive relationship exists between the investigated variables.

## 2. Materials and Methods

### 2.1. Cohort Description

This study included 293 participants (see Table 1). Eligible individuals were adults older than 18 years with primary subjective tinnitus, regardless of the presence of sensorineural hearing loss, and all participants provided informed consent before enrolment. Only participants with complete clinical, audiological, and questionnaire data were included in the analyses; therefore, no imputation of missing data was performed. Patients with secondary tinnitus or any previously diagnosed psychiatric disorders were excluded from the study. To minimise potential pharmacological confounding, patients currently receiving psychotropic medications, including antidepressants and anxiolytics, as well as medications prescribed specifically for tinnitus management that could potentially influence tinnitus perception, tinnitus severity, or psychological symptoms, were excluded from the study. Every participant received a detailed evaluation by clinicians experienced in tinnitus care, including otorhinolaryngological and audiological examinations. Written informed consent was obtained from all participants who agreed to participate in the study. The investigation was conducted in accordance with the Declaration of Helsinki and approved by the Hungarian ETT TUKEB (approval number: BM/29805–3/2025, approval date: 11 March 2026).

### 2.2. Audiological Assessments

Prior to audiological testing, each patient underwent a comprehensive otorhinolaryngological evaluation that included otoscopy, tympanometry, and acoustic reflex measurements. Possible causes of conductive hearing impairment were systematically excluded during these examinations. Subsequently, pure-tone audiometry and tinnitus matching procedures were performed for each participant by a trained audiological assistant. Pure-tone hearing thresholds were assessed in a sound-treated booth using a GSI Pello Clinical Audiometer (Grason Stadler, Inc., Milford, CT, USA). Both bone conduction (250–4000 Hz) and air conduction (125–8000 Hz) measurements were taken. Masked bone-conduction testing was carried out whenever clinically required. Pitch and loudness matching procedures were carried out for all participants with tinnitus. The pitch matching procedure evaluated frequencies between 125 and 8000 Hz, beginning with a 1000 Hz stimulus. Participants were instructed to report whether the perceived tinnitus frequency was lower or higher than the presented auditory stimulus. The stimulus frequency was progressively modified until the participant identified the closest match to their tinnitus sound. After determining the matched frequency, tinnitus intensity was assessed in 1 dB steps using the identified pitch. Tinnitus pitch and loudness values were subsequently documented manually on the audiograms.

### 2.3. Self-Reported Questionnaires

#### 2.3.1. Tinnitus Handicap Inventory (THI)

The THI evaluates the impact of tinnitus on daily life through functional (*F*), emotional (*E*), and catastrophic (*C*) subscales. The functional domain assesses social engagement and routine activities, the emotional domain addresses symptoms such as anxiety and depression, and the catastrophic domain reflects feelings related to loss of control. Each item is answered using the response options ‘yes’ (4 points), ‘sometimes’ (2 points), or ‘no’ (0 points). Total THI scores are obtained by summing the scores across the three subscales. According to the overall score, tinnitus-related handicap can be divided into five categories: no handicap (0–16 points), mild (18–36 points), moderate (38–56 points), severe (58–76 points), and catastrophic (78–100 points) [12]. The THI questionnaire was completed in Hungarian by all participants.

#### 2.3.2. Beck Depression Inventory (BDI)

To assess depressive symptom severity, the abbreviated 13-item form of the BDI was applied, as its validity has previously been confirmed in Hungarian populations [13]. Each item is rated on a four-point scale ranging from 0 (‘not at all’) to 3 (‘very much’), resulting in a highest possible total of 39 points. Total scores between 0 and 5 were interpreted as the absence of depressive symptoms, values from 6 to 11 reflected mild depression, scores of 12–15 represented moderate depression, whereas scores ≥ 16 indicated severe depressive symptoms.

#### 2.3.3. Generalised Anxiety Disorder-7 (GAD-7)

To investigate anxiety-related symptoms, all study participants filled in the GAD-7 questionnaire. This instrument contains seven questions intended to evaluate the frequency and intensity of anxiety and excessive worrying. Each item is rated on a four-point scale ranging from 0 (‘not at all’) to 3 (‘very much’), resulting in a highest possible total of 21 points. Overall scores ranging from 0–4 correspond to minimal or absent anxiety, 5–9 to mild anxiety, 10–14 to moderate anxiety, and 15–21 to severe anxiety symptoms [14]. The Hungarian-language version of the questionnaire was administered to all participants.

### 2.4. Tinnitus Characteristics

A visual analogue scale ranging from 0 to 10 was used to evaluate tinnitus loudness, pitch, complexity, and sound intolerance. For tinnitus loudness, a score of 0 represented a barely noticeable sound, whereas 10 reflected extremely loud tinnitus. Participants were asked to rate how bothersome or irritating they found the pitch (tone) of their tinnitus using a visual analogue scale from 0 to 10. On this scale, 0 indicated that the perceived pitch was not bothersome at all, while 10 signified that it was extremely bothersome. It is important to distinguish this subjective rating from the psychoacoustically matched tinnitus frequency (measured in Hz), which was analysed separately. The complexity of tinnitus refers to the variety of sounds the sufferer hears, where 0 represents only one sound and 10 represents more than 10 different sound types. Participants also rated their sensitivity to environmental sounds using the same scale.

### 2.5. Statistical Analysis

Statistical analyses were carried out using IBM SPSS Statistics version 25.0 (IBM Corporation, Armonk, NY, USA). Data normality was evaluated with the Shapiro–Wilk test, and the results demonstrated that the variables were not normally distributed. Consequently, continuous variables were summarised using medians and interquartile ranges (IQRs). Associations between variables were evaluated with Spearman’s rank correlation analysis. Spearman rank correlations were calculated between the 14 study variables. To control for multiple testing, Bonferroni correction was applied, resulting in an adjusted significance threshold of *p* < 0.00055 (0.05/91). Furthermore, linear regression analysis was performed to examine predictive relationships among the variables. Statistical significance was defined at a threshold of *p* < 0.05. A sample size calculation was conducted in advance, based on a two-tailed correlation analysis with a significance level of α = 0.05 and a statistical power of 80% (1 − β = 0.80). Assuming a small-to-moderate effect size (*r* = 0.20), the minimum required sample size was determined to be 193 participants. The final study cohort included 293 patients, which exceeded the required sample size and provided sufficient statistical power for the planned correlation analyses.

## 3. Results

Table 1 summarises the main demographic and clinical characteristics of the study population.

**Table 1:** Demographic and clinical characteristics of the study cohort. IQR = interquartile range, Q1 = lower quartile, Q3 = upper quartile, THI = Tinnitus Handicap Inventory.

Category	Variable Data
Age (median years; IQR, Q1–Q3)	50 (19; 42–61)
Sex (men/women)	114/179
Duration of tinnitus (median months; IQR, Q1–Q3)	18 (35; 5–36)
Tinnitus location	
right-sided, *n* (%)	77 (26.3%)
left-sided, *n* (%)	91 (31%)
bilateral involvement, *n* (%)	125 (42.7%)
THI	
no handicap, *n* (%)	70 (23.9%)
mild handicap, *n* (%)	95 (32.4%)
moderate handicap, *n* (%)	70 (23.9%)
severe handicap, *n* (%)	40 (13.6%)
catastrophic handicap, *n* (%)	18 (6.2%)

Table 1 shows that the majority of participants were middle-aged, with a slight predominance of females. Most cases reported a long duration of symptoms, indicating chronic conditions. The most common manifestations included left-sided and bilateral tinnitus. The total scores on the THI most frequently fell into the categories of mild and moderate impairments.

**Figure 1:**
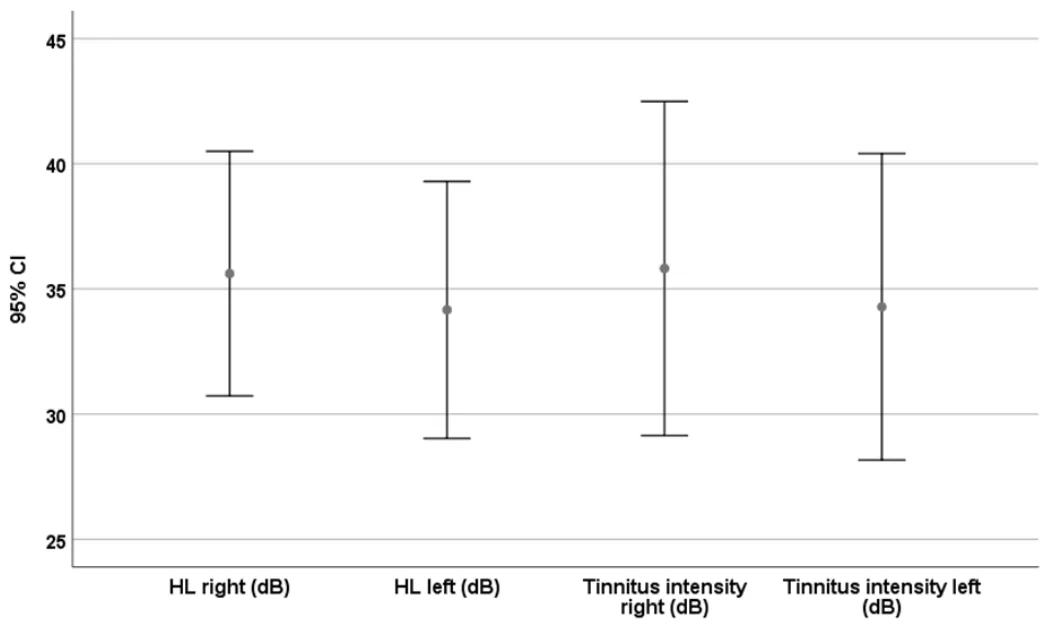
Participants’ mean hearing levels (in the 125–8000 Hz frequency range) and tinnitus intensities. dB = decibel, CI = confidence interval, HL = hearing level. Dots represent mean values, and error bars indicate 95% CIs.

Figure 1 illustrates the hearing levels (HLs) and tinnitus intensities of the study population.

A median HL of 40 dB (IQR: 30; range: 20–50) was recorded on the right side, while the left side showed a median HL of 30 dB (IQR: 30; range: 20–50). These results suggest that the majority of participants experienced moderately severe sensorineural hearing impairment. A similar pattern was observed regarding tinnitus intensities, with a median tinnitus intensity of 30 (IQR: 25; range: 20–45) on both the right and left sides (see Figure 1).

**Figure 2:**
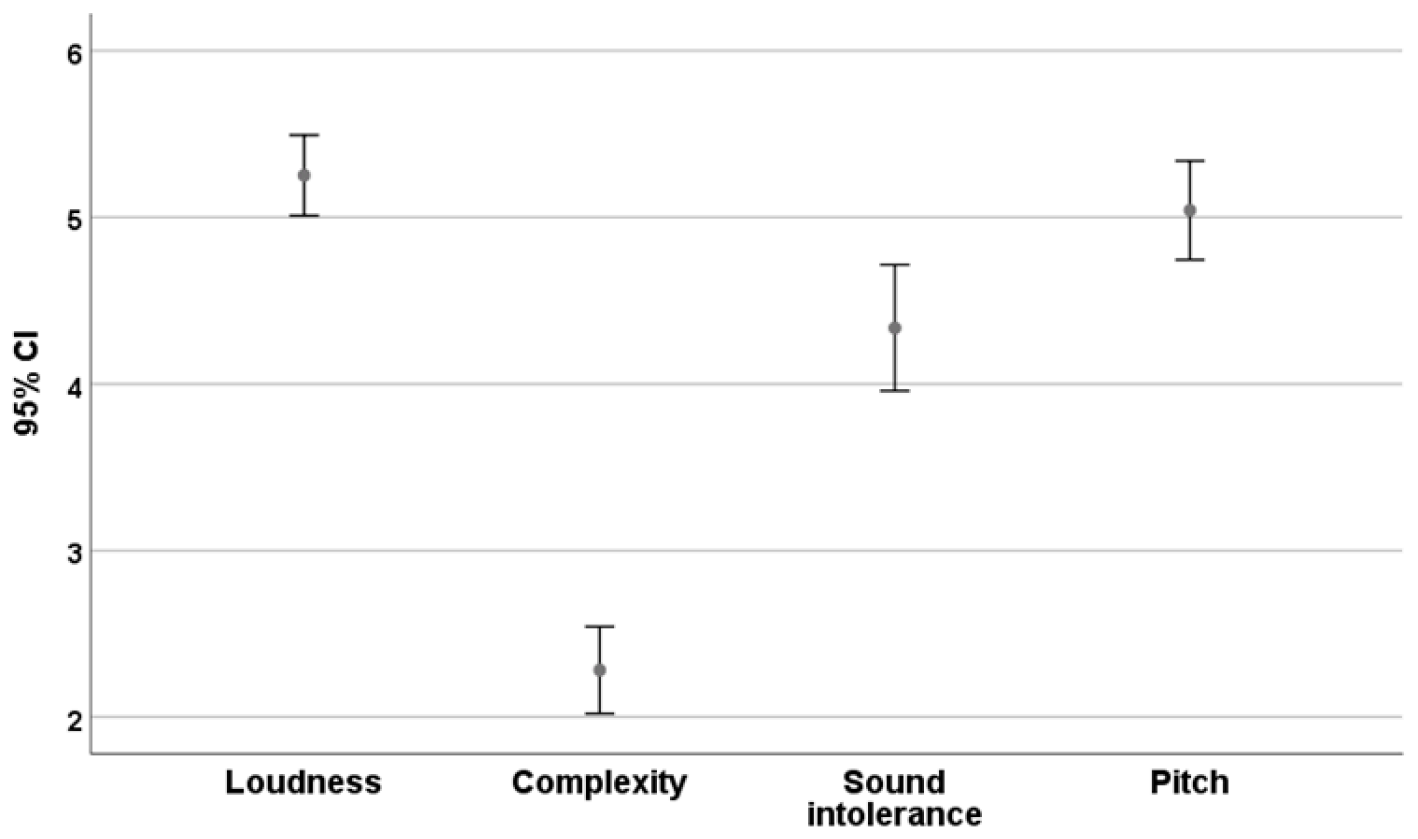
The distribution of answers regarding tinnitus characteristics on a scale from 1 to 10. CI = confidence interval. Dots represent mean values, and error bars indicate 95% CIs.

Figure 2 illustrates the characteristics of tinnitus, including loudness, pitch, complexity, and sound intolerance, on a scale from 1 to 10.

Figure 2 demonstrates that the highest median ratings were observed for tinnitus loudness (median: 5; IQR: 3, 4–7) and tinnitus pitch (median: 5; IQR: 3, 4–7), while sound intolerance also showed relatively elevated values (median: 4; IQR: 6, 1–7). By comparison, tinnitus complexity received somewhat lower scores (median: 4; IQR: 6, 1–7).

**Figure 3:**
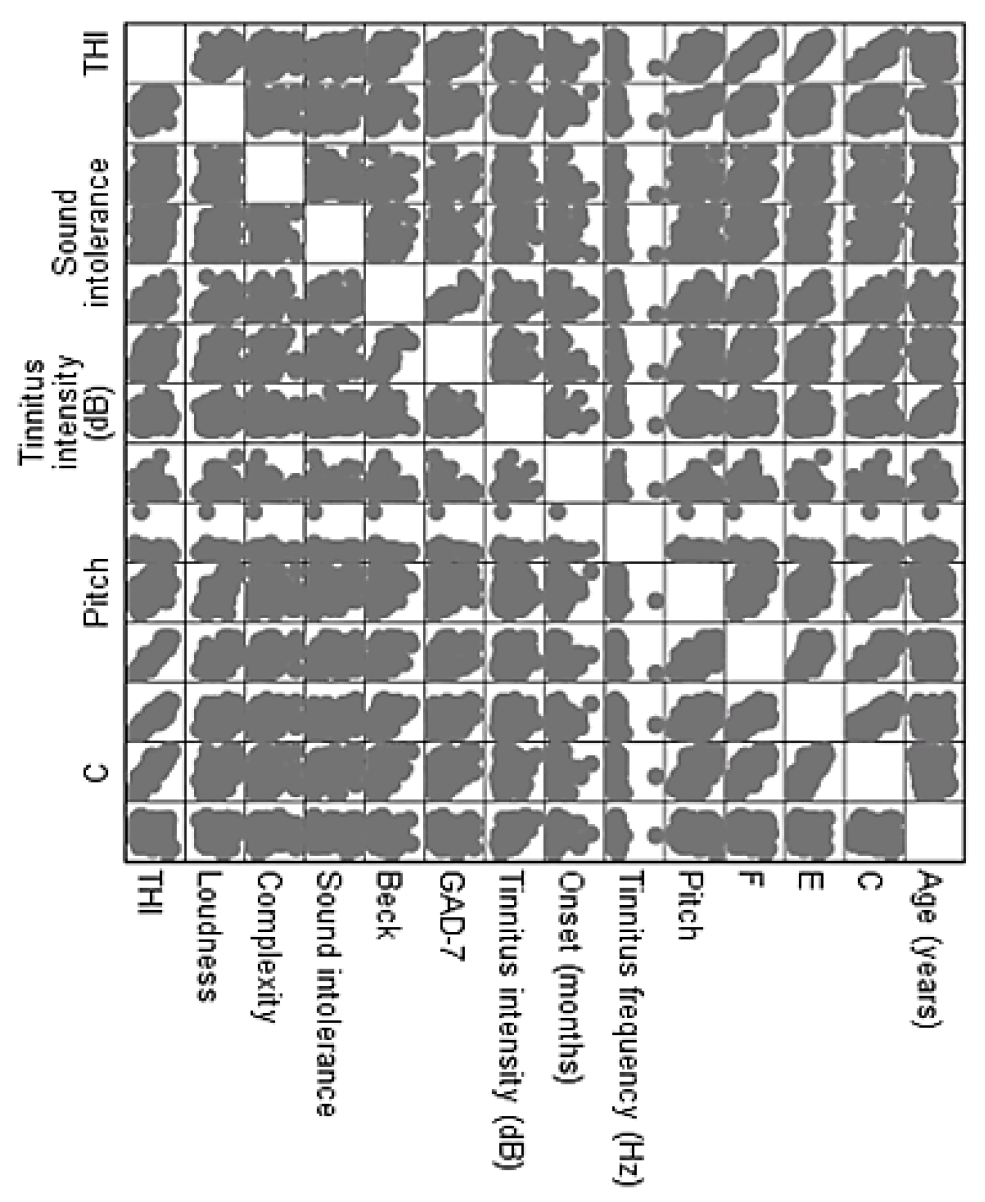
A correlation matrix illustrating the relationships between tinnitus-related variables and the results of questionnaires. *C* = catastrophic, dB = decibel, *E* = emotional, *F* = functional, GAD-7 = Generalised Anxiety Disorder-7, Hz = Hertz, THI = Tinnitus Handicap Inventory.

In the subsequent stage of the analysis, associations between tinnitus-related characteristics and the THI, BDI, and GAD-7 scores were examined (Table 2, Figure 3). Please note that, in Table 2, only the significant correlations have been included, while the results of the non-significant correlations are available in Supplementary Table S1.

Significant positive associations were identified between total THI scores and multiple tinnitus-related characteristics, including loudness (rho = 0.563, *p* = 0.000*), pitch (rho = 0.647, *p* = 0.000*), complexity (rho = 0.282, *p* = 0.000*), and intolerance to sound (rho = 0.349, *p* = 0.000*). These findings suggest that increased tinnitus loudness, higher pitch perception, and greater sound sensitivity are linked to more severe tinnitus-related disability. In addition, tinnitus severity demonstrated statistically significant positive relationships with both depressive symptoms (rho = 0.426, *p* = 0.000*) and anxiety levels (rho = 0.425, *p* = 0.000*). Collectively, these results highlight a close association between tinnitus burden and accompanying psychological symptoms. Tinnitus intensity values obtained through tinnitus matching demonstrated a significant positive association with subjectively perceived tinnitus loudness (rho = 0.232, *p* = 0.013). In contrast, no statistically significant relationship was observed between tinnitus intensity and overall THI scores (rho = 0.154, *p* = 0.100). Nevertheless, the functional (*F*) subscale of the THI revealed a significant positive correlation with tinnitus intensity measurements (rho = 0.216, *p* = 0.021). The duration of tinnitus symptoms showed significant positive associations with both perceived tinnitus loudness (rho = 0.187, *p* = 0.001) and participant age (rho = 0.209, *p* = 0.000*). These findings imply that, as people age, they are likely to experience a longer duration of tinnitus symptoms, and those with longer-lasting symptoms tend to report higher levels of subjective tinnitus loudness. Participant age demonstrated significant positive associations with both perceived tinnitus loudness (rho = 0.145, *p* = 0.013) and tinnitus intensity levels (rho = 0.510, *p* = 0.000*). In addition, a significant inverse association was identified between age and tinnitus frequency (rho = –0.295, *p* = 0.001), indicating that older individuals were more likely to report lower-frequency tinnitus.

There were notable correlations observed between tinnitus parameters and psychiatric symptoms. Total scores on the BDI showed statistically significant positive correlations with various tinnitus characteristics: loudness (rho = 0.196, *p* = 0.001), pitch (rho = 0.250, *p* = 0.000*), complexity (rho = 0.214, *p* = 0.000*), and sound intolerance (rho = 0.338, *p* = 0.000*). Similarly, scores from the GAD-7 also demonstrated significant positive correlations with these same tinnitus parameters (Table 2, Figure 3).

Due to the performance of multiple correlation analyses, Bonferroni corrections were applied. After these corrections, the correlations between tinnitus intensity and subjective tinnitus loudness, tinnitus intensity and the THI *F* subscale, tinnitus duration and subjective tinnitus loudness, subjective tinnitus loudness and BDI scores, subjective tinnitus loudness and GAD-7 scores, as well as age and subjective tinnitus loudness and tinnitus frequency, were no longer statistically significant. However, the other results remained significant (see Table 2).

In the final step of the analyses, a linear regression model was constructed (Table 3).

In the linear regression model, the total THI scores were significantly predicted by sound intolerance (*p* = 0.049*), tinnitus pitch (*p* = 0.001*), and total BDI scores (*p* = 0.048*). The total BDI scores (*p* = 0.048*) and sound intolerance (*p* = 0.049*) were both predicted by the total THI scores. These findings demonstrate that tinnitus severity significantly contributed to the simultaneous presence of psychiatric symptoms and heightened sound intolerance.

## 4. Discussion

The present investigation explored the associations among tinnitus loudness, pitch, complexity, sound intolerance, and psychological symptoms in a cohort of 293 individuals with primary subjective tinnitus. The present study demonstrates that tinnitus-related handicap is closely associated with both the perceptual characteristics of tinnitus and psychological distress. Greater tinnitus severity remained robustly associated with perceived loudness, pitch, complexity, sound intolerance, and symptoms of depression and anxiety, even after Bonferroni correction. The correlations between tinnitus intensity and subjective tinnitus loudness, tinnitus intensity and the THI-F subscale, tinnitus duration and subjective tinnitus loudness, and subjective tinnitus loudness and BDI and GAD-7 scores did not remain statistically significant after adjustment for multiple comparisons. In addition, the correlations between age and subjective tinnitus loudness and between age and tinnitus frequency were no longer statistically significant after correction. These findings suggest that the strongest and most reliable correlates of tinnitus burden are its perceptual characteristics and accompanying psychological symptoms, whereas several weaker associations should be interpreted with caution. These findings further support existing evidence highlighting the multidimensional characteristics of tinnitus and its substantial psychological burden.

THI scores demonstrated positive correlations with subjective parameters such as tinnitus loudness, pitch, complexity, and sound intolerance. The most significant correlation was found between THI and tinnitus pitch, suggesting that how patients perceive the sound of their tinnitus may have a greater impact on their handicap than the loudness of the sound itself. While tinnitus loudness is typically considered a key factor in determining distress, previous studies have indicated that it does not fully explain the severity of the handicap on its own [15,16]. Our findings suggest that higher perceived tinnitus pitch is associated with greater tinnitus-related handicap and poorer self-reported quality of life. In this context, while complexity was statistically significant, it showed a comparatively weaker correlation. This may relate to the idea of tinnitus as a heterogeneous disorder with multiple subtypes. As Mohan et al. proposed, tinnitus can exist along a continuum of severity, where increased symptom severity is associated with greater complexity in neural and biomarker activity [17]. Therefore, the way tinnitus is perceived may be associated with patterns of abnormal neural activity within the auditory pathways, although this study cannot directly evaluate these mechanisms.

The characteristics of tinnitus and the THI showed a significant correlation with depression and anxiety, as measured by the BDI and the GAD-7. This finding supports the hypothesis that tinnitus often coexists with psychological distress and a reduced quality of life. A recent meta-analysis reported that people with tinnitus are more likely to experience depression, anxiety, stress, insomnia, and suicidal ideation. These findings emphasise the need for routine psychological evaluation in patients affected by tinnitus [18]. Another investigation demonstrated a significant association between tinnitus-related distress and the severity of depressive symptoms among individuals with chronic tinnitus [19]. Similarly, our results support these findings by demonstrating that not only tinnitus severity, but also sound intolerance and pitch, can be associated with emotional burden.

Although an unadjusted analysis suggested a positive association between psychoacoustically matched tinnitus intensity and perceived loudness, this relationship was no longer statistically significant after correction for multiple comparisons. Consequently, the findings provide only limited evidence that tinnitus matching reflects patients’ subjective loudness perception. However, Vajsakovic et al. [20] noted that the subjective nature of tinnitus characterisation can complicate the reliability of its measurement. Interestingly, overall THI scores did not demonstrate a statistically significant association with objectively measured tinnitus intensity levels. The objectively measured dB level of a matched tinnitus tone may not accurately predict the real-world impact experienced by the patient. By comparison, the functional component of the THI demonstrated a statistically significant positive association with tinnitus intensity. This result suggests that the emotional impact of tinnitus is connected to specific functional limitations, including difficulties with concentration, sleep, and social interactions. This observation is consistent with the findings reported by Manning et al. [21], who showed that psychoacoustic assessments of tinnitus perception are not significantly associated with tinnitus burden measured using subjective outcome questionnaires. Furthermore, self-reported loudness tends to reflect the patient’s reaction to tinnitus, rather than serving as a purely perceptual measure.

Sound intolerance has been linked to both tinnitus-related difficulties and psychiatric symptoms. A positive correlation was found between sound intolerance and THI scores, as well as between sound intolerance and the BDI and GAD-7 scores. In linear regression analyses, sound intolerance was significantly associated with total THI scores, and higher THI scores were likewise significantly associated with greater sound intolerance. This association suggests that sound intolerance and tinnitus-related distress frequently coexist; however, the cross-sectional design does not allow conclusions regarding the direction or causality of this relationship. Furthermore, it suggests that reduced tolerance to sound is an important clinical feature commonly observed among patients with tinnitus. Jafari et al. [7] have explained that long-term exposure to occupational noise can lead to tinnitus and sound intolerance, which can subsequently affect mental health. Furthermore, a recent study [22] found that hyperacusis is significantly linked to both anxiety and depression in patients who suffer from vestibular migraines and tinnitus, regardless of their hearing thresholds. These results indicate that sound intolerance represents a clinically relevant feature that should be carefully assessed during the evaluation of patients with tinnitus.

After correction for multiple testing, only the association between age and psychoacoustically matched tinnitus intensity remained statistically significant. The initially observed associations between age and subjective loudness or tinnitus frequency did not remain significant and should therefore be interpreted cautiously. This age-related increase in tinnitus intensity is likely influenced by the progressive cochlear degeneration typical of presbycusis [23]. It is important to note that, although age seems to increase the perceived loudness of tinnitus, some studies have found no direct correlation between the extent of hearing loss due to presbycusis and the specific pitch or loudness of the phantom sound [24]. This paradox implies that changes in central auditory processing, rather than merely elevated peripheral thresholds, may drive the age-related intensification of tinnitus. Our analysis identified a significant inverse association between participant age and tinnitus pitch. Specifically, older patients tend to report lower-pitched tinnitus. This finding aligns with the typical audiometric profile of presbycusis, which often starts with high-frequency sensorineural hearing loss. As presbycusis progresses, it can also affect lower frequencies, including those relevant to speech. Multiple earlier investigations have reported an association between tinnitus pitch and the frequencies affected by sensorineural hearing impairment [25,26]. This observation has important implications for the optimisation of hearing aid fitting in patients with tinnitus [27].

Although tinnitus duration showed an unadjusted association with perceived loudness, this relationship was not retained after Bonferroni correction, suggesting only limited evidence that chronicity alone contributes to increased perceived loudness. Gudwani et al. [28] also demonstrated significant associations between high-frequency hearing thresholds and both the loudness of tinnitus and its duration of onset. They concluded that changes in hearing thresholds and the auditory pathway are connected to increases in these parameters. Longer tinnitus duration was associated with greater perceived tinnitus loudness in the present cohort. This phenomenon may be related to maladaptive neuroplastic alterations developing within the central auditory system over prolonged periods [29]. However, the relatively modest correlation coefficients suggest that tinnitus duration alone is not strongly associated with tinnitus characteristics. Rather, variations in coping strategies and levels of psychological resilience may substantially influence the nature of this relationship.

As a result, assessing tinnitus should not be confined to just audiometric testing or loudness matching. It should also incorporate evaluations of the associated handicap, as well as symptoms of depression, anxiety, and sound intolerance. Patients who score high on the THI and exhibit elevated sound intolerance may require more intensive, multidisciplinary care.

Several limitations should be acknowledged. First, the data on tinnitus and psychological characteristics were gathered through self-report measures, which are inherently subjective and may be affected by response bias. Second, the study cohort consisted of patients with primary subjective tinnitus recruited from a clinical setting, which may reduce the applicability of the findings to the general population. Another limitation is that the study cohort consisted of consecutive patients with primary subjective tinnitus recruited from a single tertiary referral centre. Consequently, the findings may not be generalisable to broader or more diverse tinnitus populations. Rather than representing a unique or highly selected cohort, this study provides additional regional clinical data on the associations between tinnitus characteristics, psychological symptoms, and tinnitus-related handicap. Therefore, the findings should be interpreted as contributing to the existing body of evidence rather than establishing novel population-level conclusions. Another limitation of this study is the lack of comparison groups, such as healthy individuals or patients with hearing loss who do not have tinnitus. As a result, we were unable to differentiate the independent effects of tinnitus, hearing impairment, and sound intolerance on the clinical and psychological associations observed. Future studies that include appropriate control groups are necessary to better understand the relative impacts of these factors. Additional adjusted regression models and subgroup analyses based on hearing loss status and tinnitus duration were not conducted due to insufficient sample size, which could not reliably support multivariable or stratified analyses. Implementing these approaches would have increased the risk of model instability, multicollinearity, and reduced statistical power. Future multicentre studies with larger cohorts are necessary to explore these subgroup-specific effects. Finally, although the study included tinnitus matching and questionnaire-based evaluations, it did not incorporate longitudinal follow-up or objective neurophysiological measures that could provide further insights into the mechanisms underlying these associations.

## 5. Conclusions

Our findings demonstrate that tinnitus-related handicap is strongly associated with perceived tinnitus loudness, pitch, complexity, sound intolerance, and symptoms of depression and anxiety. Following Bonferroni correction for multiple comparisons, the most robust associations were observed between tinnitus-related handicap and tinnitus pitch, tinnitus complexity, sound intolerance, and psychological symptoms. In contrast, several weaker associations involving subjective tinnitus loudness, tinnitus duration, and age did not remain statistically significant after correction and should therefore be interpreted with caution. These findings highlight the multidimensional nature of tinnitus and support the routine assessment of both perceptual tinnitus characteristics and psychological symptoms during the clinical evaluation of individuals with tinnitus.

## Figures and Tables

**Table 2:** Spearman correlation coefficients and corresponding *p*-values describing the associations between tinnitus-related variables and questionnaire outcomes. BDI = Beck Depression Inventory, dB = decibel, *F* = functional GAD-7 = Generalised Anxiety Disorder-7, Hz = Hertz, THI = Tinnitus Handicap Inventory. Statistical significance was defined as *p* < 0.05, with a Bonferroni-corrected significance threshold of *p* < 0.00055. Only statistically significant correlation results are presented in the table.

Factor	Rho	*p*-Value	Significant After Bonferroni Correction?
THI Loudness Pitch Complexity Sound intolerance Depression (BDI) Anxiety (GAD-7)	0.563 0.647 0.282 0.349 0.426 0.425	0.000 0.000 0.000 0.000 0.000 0.000	yes yes yes yes yes yes
Tinnitus intensity (dB) Loudness *F* subscale	0.232 0.216	0.013 0.021	no no
Tinnitus onset (months) Loudness Age (years)	0.187 0.209	0.001 0.000	no yes
Depression (BDI) Loudness Pitch Complexity Sound intolerance	0.196 0.250 0.214 0.338	0.001 0.000 0.000 0.000	no yes yes yes
Anxiety (GAD-7) Loudness Pitch Complexity Sound intolerance	0.128 0.233 0.208 0.308	0.029 0.000 0.000 0.000	no yes yes yes
Age (years) Tinnitus loudness Tinnitus intensity (dB) Tinnitus frequency (Hz)	0.145 0.510 –0.295	0.013 0.000 0.001	no yes no

**Table 3:** Using a linear regression model to analyse factors affecting total THI, BDI scores, and sound intolerance. BDI = Beck Depression Inventory, Std. = standard, THI = Tinnitus Handicap Inventory. The model has been adjusted to account for age, sex, and hearing levels. Statistical significance was defined at *p* < 0.05. Only statistically significant correlations are presented in the table summary.

	Unstandardised Coefficients		Standardised Coefficients	*t*	*p*-Value
	*β*	Std. Error	*β*		
Dependent: total THI scores					
Sound intolerance	1.024	0.514	0.144	1.993	0.049
Tinnitus Pitch	2.886	0.865	0.309	3.335	0.001
BDI	1.117	0.558	0.218	2.004	0.048
Dependent: total BDI scores					
THI	0.033	0.017	0.171	2.004	0.048
Dependent: Sound intolerance					
THI	0.036	0.018	0.255	1.993	0.049

## Data Availability

The datasets generated during and/or analysed during the current study are available from the corresponding author on reasonable request.

## Supplementary Materials

The following supporting information can be downloaded at: https://www.mdpi.com/article/10.3390/jcm15166382/s1, Table S1: Spearman correlation coefficients and corresponding *p*-values for non-significant correlations that describe associations between tinnitus-related variables and questionnaire outcomes. BDI = Beck Depression Inventory, *C* = catastrophic, dB = decibel, *E* = emotional, *F* = functional, GAD-7 = Generalised Anxiety Disorder-7, Hz = Hertz, THI = Tinnitus Handicap Inventory. Statistical significance was defined as *p* < 0.05.

## Abbreviations

The following abbreviations are used in this manuscript:

BDI	Beck Depression Inventory
C	Catastrophic
CI	Confidence interval
dB	Decibel
E	Emotional
F	Functional
GAD-7	Generalised Anxiety Disorder-7
HL	Hearing level
Hz	Hertz
IQR	Interquartile range
Q1	First quartile
Q3	Third quartile
Std.	Standard
THI	Tinnitus Handicap Inventory
