# Supplementary material for: The Relationships Among Tinnitus Intensity, Complexity, Sound Intolerance, and Psychiatric Symptoms
Source: J Clin Med. 2026 Aug 18;15(16):6382. doi: 10.3390/jcm15166382 (PMC13513216; doi:10.3390/jcm15166382)
Supplement: Supplementary file 1 [file jcm-15-06382-s001.zip › jcm-4462988-supplementary.pdf]

**Supplementary Table S1.** Spearman correlation coefficients and corresponding p-values for non-significant correlations that describe associations between tinnitus-related variables and questionnaire outcomes. BDI = Beck Depression Inventory, *C* = catastrophic, dB = decibel, *E* = emotional, *F* = functional, GAD-7 = Generalised Anxiety Disorder-7, Hz = Hertz, THI = Tinnitus Handicap Inventory. Statistical significance was defined as *p*<0.05.

| Factor | *Correlated* variable | rho | *p*-value |
|---|---|---|---|
| **THI** | Tinnitus intensity (dB) | 0.154 | 0.100 |
| | Tinnitus onset (months) | 0.041 | 0.482 |
| | Tinnitus frequency (Hz) | –0.017 | 0.856 |
| | Age (years) | 0.014 | 0.808 |
| **Loudness** | Tinnitus frequency (Hz) | –0.039 | 0.681 |
| **Complexity** | Tinnitus intensity (dB) | 0.103 | 0.275 |
| | Tinnitus onset (months) | 0.043 | 0.462 |
| | Tinnitus frequency (Hz) | –0.079 | 0.402 |
| | Age (years) | 0.068 | 0.245 |
| **Sound intolerance** | Tinnitus intensity (dB) | 0.129 | 0.169 |
| | Tinnitus onset (months) | –0.029 | 0.627 |
| | Tinnitus frequency (Hz) | –0.050 | 0.593 |
| | Age (years) | –0.014 | 0.815 |
| **Depression (BDI)** | Tinnitus intensity (dB) | 0.038 | 0.685 |
| | Tinnitus onset (months) | –0.031 | 0.596 |
| | Tinnitus frequency (Hz) | –0.121 | 0.195 |
| | Age (years) | 0.000 | 0.997 |
| **Anxiety (GAD-7)** | Tinnitus intensity (dB) | –0.053 | 0.574 |
| | Tinnitus onset (months) | –0.053 | 0.365 |
| | Tinnitus frequency (Hz) | –0.094 | 0.318 |
| | Age (years) | –0.082 | 0.160 |
| **Tinnitus intensity (dB)** | THI | 0.154 | 0.100 |
| | Complexity | 0.103 | 0.275 |
| | Sound intolerance | 0.129 | 0.169 |
| | Depression (BDI) | 0.038 | 0.685 |
| | Anxiety (GAD-7) | –0.053 | 0.574 |
| | Tinnitus onset (months) | 0.084 | 0.374 |
| | Tinnitus frequency (Hz) | –0.031 | 0.739 |
| | Pitch | 0.124 | 0.187 |
| | *E* | 0.120 | 0.202 |
| | *C* | 0.087 | 0.355 |
| **Tinnitus onset (months)** | THI | 0.041 | 0.482 |
| | Complexity | 0.043 | 0.462 |
| | Sound intolerance | –0.029 | 0.627 |
| | Depression (BDI) | –0.031 | 0.596 |
| | Anxiety (GAD-7) | –0.053 | 0.365 |
| | Tinnitus intensity (dB) | 0.084 | 0.374 |
| | Tinnitus frequency (Hz) | 0.028 | 0.764 |
| | Pitch | 0.059 | 0.314 |
| | *F* | 0.065 | 0.269 |
| | *E* | 0.043 | 0.462 |
| | *C* | 0.003 | 0.958 |
| **Tinnitus frequency (Hz)** | THI | –0.017 | 0.856 |
| | Loudness | –0.039 | 0.681 |
| | Complexity | –0.079 | 0.402 |
| | Sound intolerance | –0.050 | 0.593 |
| | Depression (BDI) | –0.121 | 0.195 |
| | Anxiety (GAD-7) | –0.094 | 0.318 |
| | Tinnitus intensity (dB) | –0.031 | 0.739 |
| | Tinnitus onset (months) | 0.028 | 0.764 |
| | Pitch | 0.057 | 0.546 |

| | | | |
|---|---|---|---|
| | *F* | –0.063 | 0.503 |
| | *E* | 0.020 | 0.834 |
| | *C* | –0.003 | 0.977 |
| **Pitch** | Tinnitus intensity (dB) | 0.124 | 0.187 |
| | Tinnitus onset (months) | 0.059 | 0.314 |
| | Tinnitus frequency (Hz) | 0.057 | 0.546 |
| | Age | 0.054 | 0.357 |
| ***F*** | Tinnitus onset (months) | 0.065 | 0.269 |
| | Tinnitus frequency (Hz) | –0.063 | 0.503 |
| | Age | 0.049 | 0.404 |
| ***E*** | Tinnitus intensity (dB) | 0.120 | 0.202 |
| | Tinnitus onset (months) | 0.043 | 0.462 |
| | Tinnitus frequency (Hz) | 0.020 | 0.834 |
| | Age | –0.015 | 0.803 |
| ***C*** | Tinnitus intensity (dB) | 0.087 | 0.355 |
| | Tinnitus onset (months) | 0.003 | 0.958 |
| | Tinnitus frequency (Hz) | –0.003 | 0.977 |
| | Age | –0.013 | 0.830 |
| **Age (years)** | THI | 0.014 | 0.808 |
| | Complexity | 0.068 | 0.245 |
| | Sound intolerance | –0.014 | 0.815 |
| | Depression (BDI) | 0.000 | 0.997 |
| | Anxiety (GAD-7) | –0.082 | 0.160 |
| | Pitch | 0.054 | 0.357 |
| | *F* | 0.049 | 0.404 |
| | *E* | –0.015 | 0.803 |
| | *C* | –0.013 | 0.830 |
